# Acupuncture-associated gastric mucosal repair and the EGF–EGFR-related signaling network: evidence, limitations, and prospects

**DOI:** 10.3389/fimmu.2026.1881531

**Published:** 2026-08-07

**Authors:** Xinyu Jiang, Jiayi Peng, Yujia Fan, Cui Li, Gang Liu

**Affiliations:** 1 Heilongjiang University of Traditional Chinese Medicine, Harbin, China; 2 Fourth Affiliated Hospital, Harbin Medical University, Harbin, China; 3 Second Affiliated Hospital, Heilongjiang University of Chinese Medicine, Harbin, China

**Keywords:** acupuncture, EGF-EGFR signaling, gastrointestinal mucosal repair, peptic ulcer, translational medicine

## Abstract

Gastric mucosal injury, particularly peptic ulcer-related injury, remains clinically relevant because incomplete or unstable repair may be followed by recurrent symptoms, bleeding, or perforation. Acid suppression and Helicobacter pylori eradication are central to treatment, but they do not directly explain the cellular and molecular processes that determine the quality of mucosal healing. The epidermal growth factor (EGF)–epidermal growth factor receptor (EGFR) network is involved in epithelial restitution, proliferation, survival, glandular reconstruction, and tissue remodeling, and therefore provides a useful framework for evaluating acupuncture-associated gastric mucosal repair. Reported acupuncture, electroacupuncture, and moxibustion studies describe changes in mucosal injury, inflammation, oxidative stress, neurohumoral regulation, perfusion-related factors, epithelial survival, and downstream signaling. However, direct receptor-centered evidence is scarce. Existing acupuncture-related evidence is largely based on total EGF or EGFR and downstream markers, rather than concurrent p-EGFR assessment, linked repair outcomes, and receptor-blocking experiments. Overall, the evidence places EGF–EGFR signaling as a plausible component of acupuncture-associated gastric repair, although receptor-dependent causality has not yet been demonstrated. Future studies should use standardized intervention protocols, time-resolved receptor and downstream measurements, causal perturbation where feasible, and clinically meaningful outcomes such as endoscopic healing, histological repair, recurrence, bleeding, and safety.

## 1 Introduction

Peptic ulcer (PU) is characterized by gastric or duodenal mucosal defects arising from an imbalance between injurious factors and mucosal defense (1, 2). Although most ulcers respond to acid suppression and H. pylori eradication, recurrence, bleeding, and perforation remain clinically important, and the global burden remains substantial (3, 4).

Current management targets injurious factors through acid suppression, H. pylori eradication, avoidance of non-steroidal anti-inflammatory drugs, and modification of recurrence-related risks. Contemporary guidelines emphasize resistance-aware eradication regimens and post-treatment confirmation (6, 7). Vonoprazan-based regimens have also expanded acid-suppression options in selected H. pylori treatment settings (8). Durable recovery, however, also depends on the quality of mucosal repair rather than apparent closure alone.

Gastric mucosal healing involves early epithelial restitution, subsequent proliferation and differentiation, microvascular recovery, glandular reconstruction, and restoration of barrier function (9). EGF–EGFR-related signaling contributes to epithelial migration, proliferation, survival, and tissue remodeling, and has broader roles in tissue homeostasis and regeneration (10–12). This network provides a practical basis for examining how early injury control may progress to stable structural and functional repair.

Acupuncture and moxibustion have been evaluated as adjunctive interventions in peptic ulcer disease (13, 14). More recent clinical and review-level evidence addresses functional gastroduodenal disorders and chronic gastritis (15–17). Reported effects include symptom modulation, changes in gastrointestinal function, reduced inflammatory and oxidative activity, and improvement in selected mucosal or repair-related indicators. These effects involve processes also regulated by growth-factor networks, although the available studies do not yet identify EGFR as the dominant upstream mediator.

This narrative review summarizes the biological context of gastric mucosal repair, evaluates the relevance of EGF–EGFR-related signaling, and classifies acupuncture-related findings according to their proximity to receptor activation. The review focuses on how total EGFR differs from receptor phosphorylation, how downstream signals should be interpreted, and which experimental and clinical studies are needed next.

## 2 Literature search and narrative synthesis approach

This article was designed as a narrative review informed by a structured literature search rather than as a systematic review or meta-analysis. The search aimed to identify representative clinical, experimental, and mechanistic studies relevant to acupuncture-related interventions, gastric mucosal injury and repair, and the EGF–EGFR-related signaling network.

PubMed/MEDLINE, Web of Science, Embase, and the Cochrane Library were searched for articles published through December 15, 2025. Search terms combined “acupuncture”, “electroacupuncture”, “moxibustion”, “EGF”, “epidermal growth factor”, “EGFR”, “phosphorylated EGFR”, “gastric mucosal injury”, “peptic ulcer”, “gastric ulcer”, “chronic gastritis”, “mucosal repair”, “mucosal healing”, “microcirculation”, “angiogenesis”, “tight junction”, “ERK/MAPK”, “PI3K/Akt”, and related terms. Reference lists of relevant reviews and original studies were also screened. Priority was given to human gastric evidence, gastric injury or ulcer models, gastrointestinal epithelial repair studies, and acupuncture research in digestive disorders.

Because the objective was mechanistic synthesis rather than estimation of a pooled intervention effect, no meta-analysis, PRISMA flow diagram, or formal study-level risk-of-bias score was produced. The narrative structure was informed by principles of transparent review reporting (18). Evidence was interpreted according to how closely each study connected receptor status with downstream signaling and tissue repair. Studies that assessed p-EGFR together with downstream phosphorylation and histological or functional outcomes provided the most direct mechanistic information. Total EGFR measurements were considered expression-level evidence rather than proof of receptor activation. Studies limited to inflammatory mediators, oxidative-stress indices, perfusion, or barrier-related proteins were used to describe the surrounding repair environment. Clinical findings were also interpreted in light of randomization, missing data, outcome measurement, and selective reporting, without assigning a formal risk-of-bias score (80).

## 3 Gastric mucosal injury and repair as the biological context

### 3.1 Key repair processes after gastric mucosal injury

Gastric mucosal injury arises when acid-peptic exposure, Helicobacter pylori-associated inflammation, drug-related impairment of mucosal defense, stress, or other injurious conditions exceed local protective capacity (19–21). Although the initiating mechanisms differ, established injury commonly involves epithelial disruption, inflammatory infiltration, impaired perfusion, and disturbance of epithelial renewal.

Repair proceeds through overlapping stages. Early epithelial restitution depends on migration of surviving cells across the denuded surface, whereas later recovery requires proliferation, lineage differentiation, glandular reconstruction, and restoration of mucosal architecture (22, 23). Human gastric gland studies further indicate that EGF-related and BMP-related signals participate in epithelial differentiation and spatial organization, while persistent H. pylori-associated inflammation may disrupt these programs (24, 25). Gastric injury may also induce lineage plasticity and metaplastic responses rather than simple expansion of a fixed progenitor population (26, 27). Stable healing additionally requires microvascular recovery and restoration of barrier function. Adequate perfusion supports tissue regeneration, whereas mucus production, epithelial continuity, and junctional organization help restore resistance to recurrent luminal injury (28).

Inflammatory-cell, barrier, and vascular responses together shape the local conditions required for mucosal repair. Representative supporting studies are summarized in **Table 1**. Each outcome reflects a different aspect of repair. Ulcer area and histological injury reflect tissue-level recovery, whereas proliferation, apoptosis, angiogenesis, and barrier-related markers describe distinct cellular or functional dimensions of healing (23).

**Table 1:** Representative supporting evidence and its evidential distance from gastric EGFR activation.

Evidence domain	Representative sources	Model or population	Main contribution	Interpretive role
Clinical disease context	(53–56)	Peptic ulcer and diagnostic care	Diagnosis, noninvasive testing, and resistance-guided therapy	Clinical context
Ulcer bleeding management	(4, 5, 81)	Acute upper-GI bleeding	Endoscopic and pharmacological management	Clinical outcome context
Gastric regeneration	(22–24)	Human gastric glands and gastric injury models	Regeneration, glandular organization, and EGF-related differentiation	Direct gastric repair biology
Gastric differentiation and plasticity	(25–27)	Gastric glands and upper-GI stem-cell models	Differentiation, lineage identity, and injury-induced plasticity	Direct gastric repair biology
Barrier physiology	(28, 31, 57, 85)	Gastric and gastrointestinal models	Permeability, mucus, trefoil factors, and epithelial continuity	Supports outcome selection
Barrier regulatory pathways	(60, 63, 68, 75)	Predominantly intestinal models	Tight-junction, IL-1β, autophagy, and uPA–uPAR regulation	Supports broader barrier biology
Inflammatory-cell regulation	(58, 59, 65, 73)	Predominantly intestinal models	TLR, macrophage, neutrophil, and innate immune responses	Supports repair-microenvironment interpretation
Alternative gastric repair	(61, 71, 74)	Gastric injury and ulcer models	Non-EGFR cytoprotection and bioactive interventions	Shows alternative repair mechanisms
Broader repair platforms	(62, 67, 69, 70)	GI and tissue-repair models	Healing frameworks, radiation injury, biomaterials, and multimodal therapy	Background repair evidence
H. pylori and host signaling	(72, 77, 78, 86)	Atrophic gastritis and H. pylori-associated disease	Inflammatory and kinase-centered pathway convergence	Disease-specific background
Acid-related disorders	(29, 30, 87, 88)	Gastric acid physiology and GERD/PPI contexts	Acid environment and treatment-associated mucosal changes	Gastric physiological context
Functional dyspepsia context	(52, 82–84, 89)	Functional dyspepsia and gastroduodenal disorders	Motility, sensation, duodenal mechanisms, and conventional management	Clinical context
Acupuncture in functional GI disorders	(15, 16, 90–92)	Functional dyspepsia	Symptoms, motility, and neuromodulatory outcomes	Supports gastrointestinal effects
Electrical stimulation and acute GI injury	(43, 44, 93)	GI motility and acute gastrointestinal injury	Neuromodulatory and inflammatory outcomes	Supports neuromodulatory interpretation
Intervention reporting standards	(45–49)	Animal and clinical research methods	Reproducibility, intervention, and sham reporting	Methodological support
Bias and outcome design	(50, 51, 80, 94, 95)	Animal and randomized clinical research	Outcome selection, bias assessment, and sham-device validation	Methodological support
Acupuncture safety	(96–99)	Systematic review and large prospective surveys	Adverse-event profile and safety reporting	Clinical implementation evidence
Foundational EGFR biology	(76, 100, 101)	General receptor and systems biology	Dimerization, autophosphorylation, and pathway cross-talk	Mechanistic framework
Gastric growth factors and angiogenesis	(102–104)	Experimental gastric ulcer and endothelial models	Growth-factor signaling, angiogenesis, and vascular reconstruction	Direct gastric repair biology

### 3.2 Model heterogeneity and interpretation of repair evidence

Different gastric injury models represent distinct biological stages. H. pylori-related models emphasize persistent inflammation and altered epithelial differentiation, NSAID-related models emphasize impaired mucosal defense, and ethanol- or stress-induced models primarily reflect acute epithelial damage, oxidative stress, and microcirculatory disturbance. Chronic ulcer models provide a longer observation window for epithelial regeneration, angiogenesis, glandular reconstruction, and the stability of healing (22).

This distinction is important when interpreting acupuncture-related evidence. Improvement in an acute model may mainly reflect anti-inflammatory, antioxidant, neurohumoral, or perfusion-related effects, whereas improvement in a chronic ulcer model provides stronger evidence of structural repair. Changes in downstream phosphorylation, inflammatory mediators, or barrier proteins should therefore be interpreted together with model type, sampling time, and histological outcome (23–25).

Other gastrointestinal injury models help identify relevant repair outcomes, although their findings require cautious application to acupuncture-related gastric injury. These indirect sources are mapped in **Table 1**.

Interpretation should account for model type, sampling time, and whether the measured outcome reflects pathway activity or tissue repair. On this basis, the following sections distinguish receptor-centered evidence from downstream pathway convergence and broader regulation of the repair microenvironment.

## 4 The EGF–EGFR axis in gastric mucosal repair

### 4.1 Relevance of EGF–EGFR signaling to mucosal repair

EGFR is a receptor tyrosine kinase that links extracellular ligand binding to intracellular programs regulating migration, proliferation, survival, and tissue remodeling (11, 12). In gastric repair, EGF-related signaling acts within a broader network of growth factors and local environmental inputs rather than as an isolated linear pathway (32, 33).

Human gastric gland studies show that EGF interacts with differentiation programs that shape glandular organization (24). Recent work on gastric regeneration and injury-induced plasticity further emphasizes restoration of epithelial identity and tissue architecture (26, 27). These findings justify examining EGFR in gastric repair, while the acupuncture-specific link remains to be defined.

Inflammation, oxidative injury, altered perfusion, and epithelial-state changes can modify receptor expression or downstream signaling (19, 23). EGFR expression is most informative when interpreted alongside receptor activation, injury context, and tissue repair.

### 4.2 Total EGFR expression is not equivalent to EGFR activation

Total EGFR reflects receptor abundance, whereas p-EGFR more directly reflects functional receptor activation after ligand binding and autophosphorylation (32, 100). Increased EGF or total EGFR may indicate involvement of the network in a regenerative response, but it does not demonstrate that the receptor is functionally active.

This distinction is supported by gastric ulcer research. Increased total EGFR expression has been reported at the ulcer margin during healing (34). Stronger receptor-centered evidence comes from experiments showing increased EGFR kinase activity and phosphorylation together with ERK activation, as well as delayed healing after pharmacological inhibition of EGFR kinase activity (35).

A more complete evidence chain should combine p-EGFR or a p-EGFR/EGFR ratio with downstream phosphorylation and structural or functional repair outcomes. Gastrointestinal epithelial studies showing that specific EGFR autophosphorylation sites influence chemotaxis and restitution further illustrate why receptor activation is more informative than receptor abundance alone (36).

### 4.3 Downstream signaling and repair-related interpretation

EGFR can engage MAPK/ERK and PI3K/Akt pathways involved in epithelial migration, proliferation, and survival (30). Gastric ulcer studies support EGFR–ERK coupling during healing (35). However, ERK, Akt, STAT3, and related pathways can also be regulated by inflammatory, neural, oxidative, and other growth-factor inputs; downstream changes therefore indicate convergence rather than receptor specificity.

Downstream phosphorylation becomes mechanistically informative when it follows p-EGFR activation in a coordinated temporal sequence and is accompanied by tissue repair. Evidence is strengthened further when pharmacological or genetic interruption of EGFR attenuates the response.

For acupuncture research, downstream changes are best understood as evidence of pathway convergence unless EGF, p-EGFR, linked downstream phosphorylation, and repair outcomes are assessed within the same study.

## 5 Acupuncture and the EGF–EGFR-related repair network

Clinical studies and evidence syntheses have examined acupuncture-related interventions in peptic ulcer disease and chronic gastritis (13, 17). Functional gastroduodenal research provides additional evidence on symptoms and neurohumoral regulation (15, 16). Direct receptor-related evidence is represented by an isolated moxibustion study (37), whereas most other findings are indirect.

### 5.1 Direct receptor-centered evidence involving EGF, EGFR, or p-EGFR

**Figure 1:**
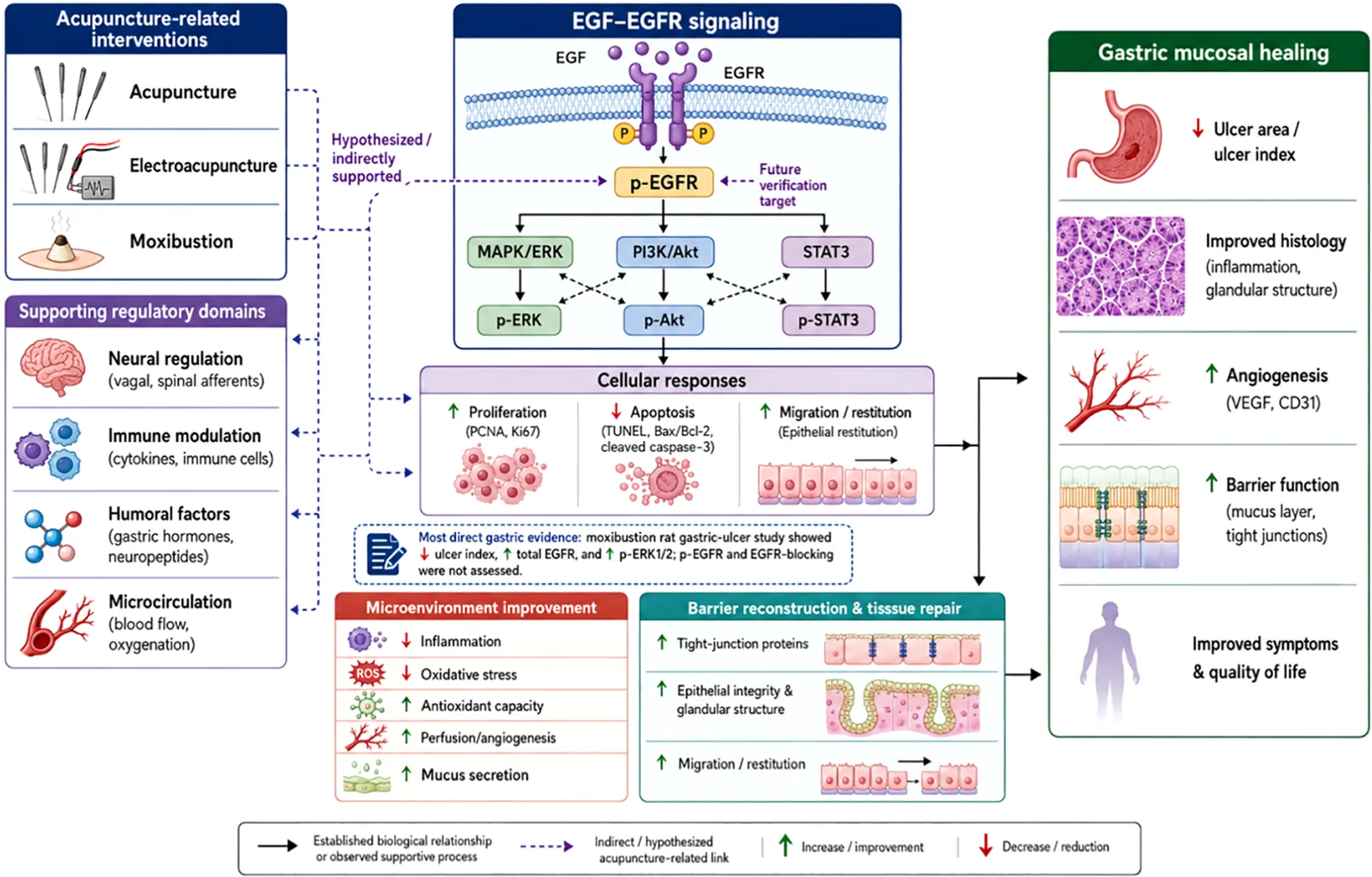
Evidence-informed framework linking acupuncture-related interventions with EGF–EGFR-related gastric mucosal repair. Solid arrows indicate established biological relationships or directly assessed links, dashed arrows indicate indirect or proposed associations, and blunt-ended lines indicate inhibition.

The potential relationships among acupuncture-associated regulation, EGF–EGFR-related signaling, cellular responses, and gastric mucosal healing are summarized in **Figure 1**.

Direct receptor-related evidence is currently based mainly on one moxibustion study. In an alcohol-induced gastric ulcer model, acupoint moxibustion reduced the ulcer index and alleviated gastric mucosal histological injury, accompanied by increased total EGFR and p-ERK1/2 expression (37). These findings support an association between moxibustion-related mucosal repair and the EGFR–ERK signaling network. However, p-EGFR was not assessed, and no EGFR inhibition or receptor-blocking experiment was performed.

The study therefore provides receptor-expression and downstream-signaling evidence, but not direct evidence of receptor activation or EGFR-dependent causality. Clinical studies in peptic ulcer disease have reported possible symptomatic or healing-related benefits of acupuncture (13, 14), although they did not assess gastric p-EGFR or link receptor activation with tissue repair.

### 5.2 Downstream pathway convergence

ERK/MAPK and PI3K/Akt are biologically relevant to epithelial migration, proliferation, and survival during mucosal repair (30). General EGFR signaling studies support their placement downstream of receptor activation (33). Gastric ulcer research provides more disease-specific evidence for EGFR–ERK coupling. The moxibustion study described above reported increased p-ERK1/2 alongside total EGFR (37), but downstream phosphorylation does not identify EGFR as the only possible upstream input.

Review-level synthesis in chronic gastritis describes modulation of repair-related signaling after acupuncture-associated interventions (17). Neuroimmune research provides plausible non-EGFR routes through which electroacupuncture may alter inflammatory signaling (38, 40). Together, these findings indicate that acupuncture may engage shared repair pathways, although the upstream receptor cannot yet be assigned.

Mechanistic confidence would increase if acupuncture-related p-EGFR changes preceded downstream activation and tissue repair, and if EGFR interruption attenuated the response. Until such studies are available, downstream signals are best used to map pathway involvement, not to assign EGFR dependence.

### 5.3 Inflammation and oxidative stress

Inflammation and oxidative stress can aggravate gastric epithelial injury, increase permeability, and impair cellular survival and restitution (19–21). These processes are important components of the repair environment but are not specific readouts of EGF–EGFR activity.

Electroacupuncture can engage anatomically defined neuroimmune pathways involved in inflammatory regulation (38). More broadly, nerve stimulation may modulate inflammation through cholinergic, catecholaminergic, splenic, and adrenal pathways (79). Reviews also describe neural, immune, and humoral signaling from acupoints to target organs, including ST36-related effects (39–41).

Chronic gastritis syntheses report reductions in inflammatory mediators and oxidative-stress indices after acupuncture-related interventions. These changes are consistent with a more favorable repair environment, although their relationship with gastric EGFR activation has not been directly tested. Broader stimulation studies identify additional neural and immune pathways, but most were not designed to evaluate gastric EGFR signaling. They are therefore summarized as indirect supporting evidence in **Table 1**. Thus, anti-inflammatory and antioxidant effects are likely to contribute to mucosal recovery, although they do not by themselves define the upstream receptor pathway.

### 5.4 Microcirculation, neurohumoral regulation, and barrier reconstruction

Adequate mucosal perfusion supports epithelial metabolism, angiogenesis, and removal of inflammatory metabolites during healing. Improved microcirculation may support healing through autonomic, endothelial, inflammatory, and growth-factor-related mechanisms.

Acupuncture may influence gastroduodenal function through autonomic regulation, brain–gut communication, gastrointestinal hormones, motility, and visceral sensitivity (16). Neuroimmune mechanisms provide a plausible route for remote organ regulation (38, 40), whereas evidence from electrical stimulation and acute gastrointestinal injury remains indirect.

Acid secretion and parietal-cell activity shape the gastric mucosal environment (29, 30). Barrier reconstruction involves restoration of epithelial continuity and resistance to luminal injury; detailed permeability evidence derives largely from broader gastrointestinal models (28, 85).

Ulcerative-colitis studies provide supporting evidence that acupuncture-related interventions can influence mucosal healing and barrier pathways (42), although direct confirmation in gastric ulcer models is still required. Functional dyspepsia studies support assessment of neuromodulatory and symptom outcomes, but not receptor-centered gastric healing. Additional clinical, guideline, and historical acupuncture evidence is summarized in **Table 1**. These findings identify relevant functional and tissue-level outcomes for future gastric p-EGFR studies.

**Table 1** summarizes contextual and indirect sources that support outcome selection or biological plausibility. Their inclusion does not imply direct acupuncture-induced EGFR activation.

### 5.5 Evidence hierarchy and proposed repair model

The evidence can be arranged in successive levels. Receptor-centered findings involving EGF, total EGFR, and especially p-EGFR are closest to the proposed mechanism. Downstream p-ERK, p-Akt, or p-STAT3 findings indicate convergence with repair-related signaling. Cellular, structural, perfusion-related, inflammatory, oxidative, and barrier outcomes describe the biological context in which repair occurs.

Each level answers a different mechanistic question. Ulcer index, histological improvement, cytokines, oxidative-stress markers, or downstream phosphorylation can support an acupuncture-associated repair effect, but none independently establishes EGF–EGFR activation. Stronger mechanistic evidence requires receptor phosphorylation, downstream activation, structural or functional repair, and preferably EGFR interruption within the same experimental framework.

**Figure 2:**
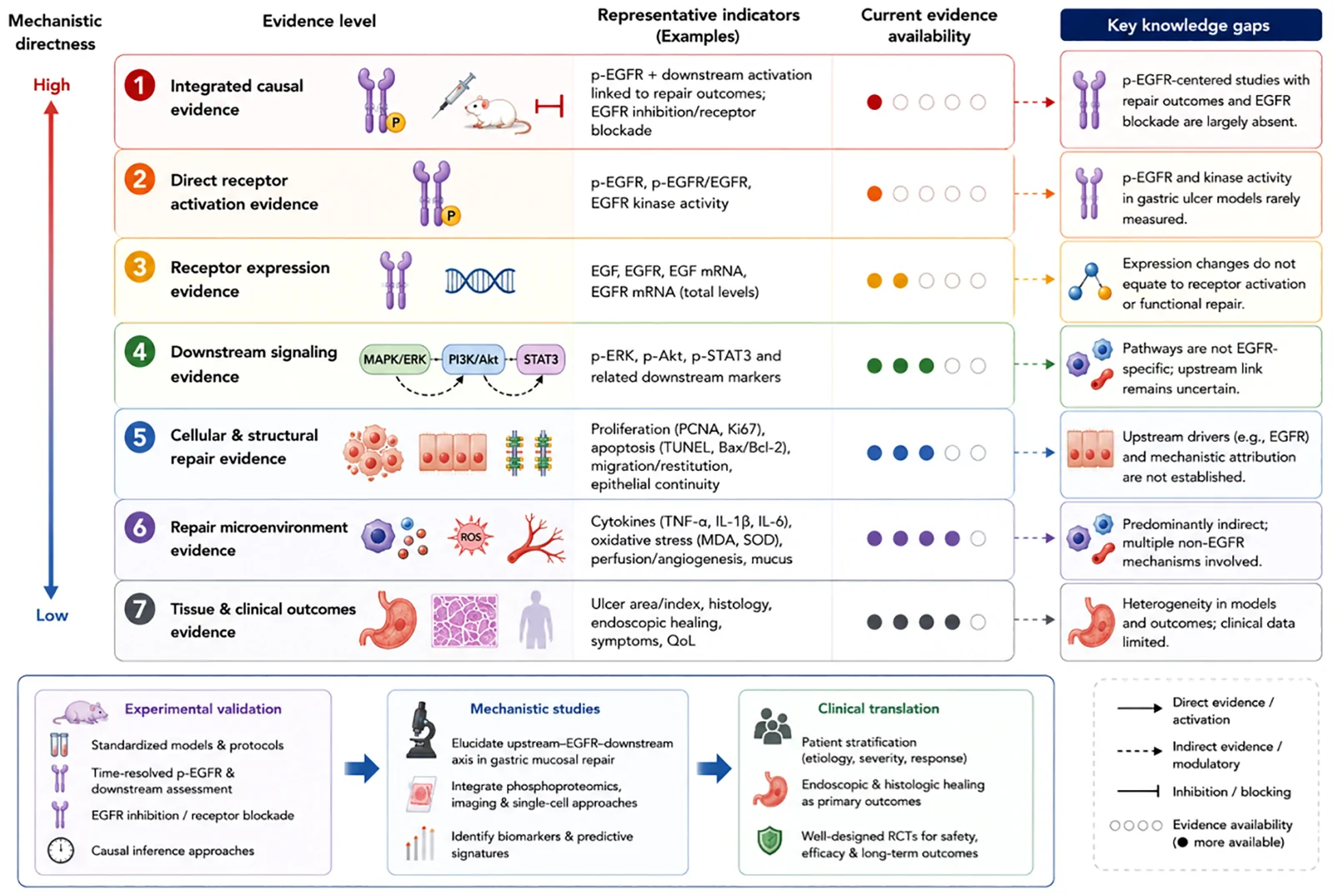
Mechanistic evidence hierarchy for acupuncture-associated gastric mucosal repair and EGF–EGFR-related signaling. Evidence levels are arranged according to mechanistic directness rather than the number of available studies. Filled circles indicate relative evidence availability. Solid arrows indicate established biological relationships or directly assessed links, dashed arrows indicate indirect or proposed associations, and blunt-ended lines indicate inhibition or blockade. Integrated causal evidence and p-EGFR-centered receptor activation remain the least available evidence levels.

Taken together, the evidence places acupuncture-associated mucosal repair within a biological network that overlaps with EGF–EGFR signaling. The direct receptor-related evidence is limited to an isolated study that measured total EGFR and p-ERK1/2 rather than p-EGFR. Neuroimmune findings mainly describe regulation of the repair microenvironment (39), while broader anti-inflammatory evidence remains non-receptor-specific (40, 41). Other gastrointestinal studies provide indirect mucosal or functional support (42). The evidence is therefore concentrated at the tissue, cellular, neuroimmune, and repair-microenvironment levels, whereas p-EGFR-centered and integrated causal evidence remains limited (**Table 2**, **Figure 2**).

**Table 2:** Evidence hierarchy and interpretive framework for acupuncture-associated EGF–EGFR-related gastric mucosal repair.

Evidence level	Representative indicators	Interpretation	Current evidence	Main gap
Integrated causal evidence	p-EGFR, downstream phosphorylation, repair outcomes, EGFR inhibition/blockade	Strongest support for an EGFR-dependent mechanism	Very scarce	Few integrated causal studies
Direct receptor activation	p-EGFR, p-EGFR/EGFR, EGFR kinase activity	Indicates functional receptor activation	Very limited	Rarely assessed in acupuncture studies
Receptor expression	EGF, total EGFR, EGF/EGFR mRNA	Suggests pathway involvement but not activation	Limited	Expression may be compensatory
Downstream signaling	p-ERK, p-Akt, p-STAT3	Indicates convergence with repair-related signaling	Limited to moderate	Not EGFR-specific
Cellular and structural repair	PCNA/Ki67, TUNEL, Bax/Bcl-2, restitution, tight-junction proteins	Reflects cellular or structural healing	Moderate	Upstream mechanism uncertain
Repair microenvironment	Cytokines, MDA, SOD, blood flow, VEGF, CD31, mucus	Reflects anti-inflammatory, antioxidant, and perfusion-related effects	Relatively common	Predominantly indirect
Tissue and clinical outcomes	Ulcer index, histology, endoscopic healing, symptoms, recurrence	Indicates injury or healing outcomes	Relatively common	Clinical and long-term data limited

The available moxibustion study assessed total EGFR and p-ERK1/2 but did not measure p-EGFR or include EGFR-blocking experiments. Therefore, it supports receptor-expression and downstream-signaling evidence rather than direct receptor activation or integrated causal evidence.

## 6 Limitations of current evidence and this review

Several features of the current evidence affect interpretation. Acupuncture-related gastric research is dominated by heterogeneous animal studies, clinical observations, and secondary syntheses, and studies combining histological healing with receptor-centered measurements remain uncommon. Differences in injury models, acupoint selection, stimulation method, intensity, treatment duration, and sampling time further limit direct comparison across studies.

Direct EGFR-related evidence remains largely restricted to total receptor expression and downstream signaling markers; p-EGFR measurements and EGFR inhibition or receptor-blocking experiments are rarely reported (34, 35). Evidence from functional gastrointestinal disorders, ulcerative colitis, and acute gastrointestinal injury provides useful biological context, but its relevance to gastric ulcer repair requires direct confirmation.

As a narrative review, this article did not undertake quantitative evidence grading or a formal risk-of-bias assessment. Incomplete reporting of randomization, blinding, exclusions, and prespecified outcomes may reduce confidence in preclinical findings (51). Contemporary studies using phosphorylation assays, spatial imaging, and causal-interference approaches are needed to extend the foundational gastric-ulcer receptor studies (34, 35).

## 7 Future directions

The next phase of research should test the full sequence from acupuncture intervention to receptor activation, downstream signaling, and structural or functional healing. Experimental validation, early clinical mechanistic studies, and standardized clinical trials represent complementary stages in this process.

### 7.1 Experimental validation of the EGF–EGFR-related repair pathway

Model choice should reflect the stage of repair under investigation. Acute ethanol or stress models are suitable for early epithelial injury and perfusion-related responses, whereas chronic ulcer models provide a longer window for regeneration, angiogenesis, glandular reconstruction, and barrier recovery.

Acupuncture protocols should report acupoint rationale, stimulation parameters, treatment schedule, and practitioner details. Animal experiments should prespecify randomization, blinding, sample-size rationale, exclusions, and outcomes. Additional reporting and sham-control guidance is listed in **Table 1**.

Mechanistic assessment should distinguish receptor abundance from activation by including total EGFR, p-EGFR, and the p-EGFR/EGFR ratio. Downstream phosphorylation should be paired with structural repair outcomes (34–36). The direct moxibustion study should be interpreted as total EGFR plus p-ERK1/2 evidence rather than p-EGFR evidence.

Receptor and signaling measurements should be paired with histological healing and epithelial continuity. Proliferation, apoptosis, angiogenesis, and barrier-related indicators may be added according to the model, but a small number of prespecified primary outcomes is preferable to a large exploratory panel (50).

Time-resolved sampling is needed to determine whether receptor phosphorylation precedes downstream activation and tissue repair or appears secondarily after injury has already improved. Time-resolved imaging approaches may help clarify the temporal relationship between pathway activation, epithelial migration, and tissue repair (64). Early time points may capture receptor and restitution responses, whereas later sampling can assess proliferation, angiogenesis, glandular reconstruction, and barrier recovery.

Causal involvement can be tested by determining whether EGFR inhibition weakens the repair response. Pharmacological EGFR inhibition, receptor-blocking approaches, or genetic interference should be used where feasible. If EGFR inhibition consistently weakens acupuncture-associated repair, the case for receptor dependence would be considerably strengthened (35, 36).

Biomarker selection also requires analytic validity. Serum or tissue EGF assays should be interpreted according to assay format and biological matrix (66).

### 7.2 Early clinical mechanistic studies

Clinical mechanistic studies should balance scientific value against the invasiveness of tissue sampling. Gastric biopsy solely for p-EGFR measurement may not be justified in every patient, but mechanistic substudies can be embedded in clinically indicated baseline or follow-up endoscopy.

Where ethically appropriate, standardized specimens from the ulcer margin or adjacent mucosa could be used to examine p-EGFR, p-ERK, p-Akt, epithelial proliferation, apoptosis, microvascular changes, and barrier-related features together with histological healing. This design would directly connect molecular changes with tissue repair in the same patient.

Serum or salivary EGF may be explored as a supplementary, less invasive marker, but it should not be treated as a surrogate for gastric receptor activation. Such measurements are more informative when interpreted alongside endoscopic and histological outcomes.

Clinical studies should standardize acupoint selection, stimulation parameters, treatment course, and practitioner qualifications. Concomitant medication, rescue therapy, adherence, and sham procedures should also be prespecified. Acupuncture is most appropriately evaluated as an adjunct to established care rather than as a replacement for acid suppression or H. pylori eradication.

Outcome selection should correspond to the hypothesis. A focused set of clearly defined primary and secondary outcomes is preferable to a broad collection of weakly related biomarkers (50). A practical early mechanistic core could include endoscopic healing, histological repair, p-EGFR-related signaling, and one or two prespecified cellular or barrier outcomes.

Sham design requires explicit justification. Non-penetrating devices developed by Streitberger and Park can improve participant blinding (94, 95), but they may not be physiologically inert. Trials should therefore assess treatment credibility, sensory experience, and the possibility that the comparator produces biological effects.

### 7.3 Standardized clinical trials and translational outcomes

Larger clinical trials should stratify or prespecify analyses according to ulcer etiology and disease stage. H. pylori-associated, NSAID-related, recurrent, and idiopathic ulcers differ in inflammatory background and recurrence risk (2). They also differ in treatment requirements and the role of eradication therapy. Active ulcers should be distinguished from healing-stage lesions.

Clinical outcomes should extend beyond symptom relief. Endoscopic healing and time to healing are important short-term outcomes, whereas recurrence, rebleeding, perforation, hospitalization, further intervention, medication use, quality of life, adherence, and adverse events are more informative for long-term value.

Follow-up should be long enough to evaluate the stability of healing. Short treatment courses may capture symptom change or initial closure but cannot determine whether structural recovery is durable or whether recurrence has been reduced.

Inflammatory and oxidative-stress markers may be included when linked to a prespecified mechanism, but they should remain secondary to clinically meaningful and tissue-level outcomes (38).

Multicenter studies will require consistent definitions of intervention, comparator, rescue medication, adherence, healing, recurrence, and adverse events. Acupuncture delivery and sham procedures should be reported in sufficient detail to permit replication.

A practical sequence would begin with receptor-focused animal studies, proceed to biopsy-based mechanistic studies, and culminate in multicenter adjunctive-treatment trials. Safety should be treated as a prespecified outcome, with systematic recording of event severity, relatedness, management, withdrawal, practitioner qualifications, and sterile-needle procedures; representative evidence is summarized in **Table 1**.

## 8 Conclusion

Acupuncture-related interventions appear to influence several processes central to gastric mucosal repair, including inflammatory control, oxidative balance, neurohumoral regulation, perfusion, epithelial survival, and downstream signaling. These effects overlap with the EGF–EGFR repair network and provide a clear basis for receptor-focused investigation. Current studies, however, have not yet defined a p-EGFR-dependent mechanism. Future work combining standardized acupuncture protocols, time-resolved receptor measurements, causal interruption, and clinically meaningful healing outcomes will determine the contribution of EGFR signaling to this repair response.
